# Non-pharmaceutical interventions (NPIs) to control influenza spread among children in primary school and kindergarten: class-suspension or symptom-based isolation?

**DOI:** 10.1186/s12879-025-10701-3

**Published:** 2025-03-06

**Authors:** Hui Xiang, Jie Zhang, Linlin Yang, Yan Wang, Tao Li, Xiaoqing Tang, Jing Qin, Wenwen Deng, Rui Zhang

**Affiliations:** 1 Nanan District Center for Disease Control and Prevention, Chongqing, Nanan China; 2 Zigong City Center for Disease Control and Prevention, Sichuan, Zigong China; 3 S.H.Ho Research Centre for Infectious Diseases, The Chinese University of Hong Kong, Sha Tin, Hong Kong, China; 4 Stanley Ho Centre for Emerging Infectious Diseases, The Chinese University of Hong Kong, Sha Tin, Hong Kong, China; 5 JC School of Public Health and Primary Care, The Chinese University of Hong Kong, Sha Tin, Hong Kong China

**Keywords:** Influenza, Class-suspension, Symptom-based isolation, Children

## Abstract

**Background:**

Non-pharmaceutical interventions (NPIs) are critical for influenza control and prevention, however, the data about such interventions are insufficient among students in grades below middle school. Hence, this study aims to explore the effectiveness of NPIs (class-suspension and symptom-based isolation) on the control of influenza spread among children in primary school and kindergarten. Findings will support evidence-based strategies for influenza control among children enrolled in primary schools and kindergartens.

**Methods:**

We purposively selected two regions (Zigong and Nanan) in Southwest China as study places, and class-suspension and symptom-based isolation was conducted in the two regions, respectively. RT (effective reproduction number) value, incidence cases, and attack rate were considered as outcome indicators. R4.1.2 software was used to conduct statistical analysis, and *p* < 0.05 (two-tailed) was considered as statistically significant.

**Results:**

In total, 593 students in the Nanan district received symptom-based isolation while 1340 students in Zigong city received class-suspension. The vaccination rate of Zigong (21.27%) was much greater than Nanan (8.26%) (*p* < 0.001). Parents in the Nanan were more highly educated, with undergraduate, master and above degrees, compared to parents in the Zigong (*p* < 0.05). Though there was no statistical significance difference in RT between Nanan (1.23) and Zigong (1.16) after quarantine and control measures were conducted, the RT value in the two regions was sharply decreased. The incident cases after class-suspension was much higher than symptom-based isolation in both kindergarten and primary school. The attack rate had a significant difference between class-suspension and symptom-based isolation in primary school (*p* < 0.05).

**Conclusions:**

Both symptom-based isolation and class-suspension are effective measures in control of influenza spread, and symptom-based isolation are more effective than class-suspension in primary school. Health education and daily surveillance are needed in the control and prevention of influenza.

**Supplementary Information:**

The online version contains supplementary material available at 10.1186/s12879-025-10701-3.

## Background

Emerging influenza epidemics, such as H7N9, HIN1, continually threaten global health and economics [1, 2]. The World Health Organization (WHO) estimated that, 5-10% of adults and 20-30% of children are infected with influenza annually, which causes 3 to 5 million cases of severe illness and 250,000 to 500,000 deaths [1]. Influenza infections account for 10% global respiratory hospitalizations in children under 18 years of age [3, 4]. For rapidly emerging novel strains of influenza outbreaks, pharmaceutical measures might be unavailable or ineffective, and non-pharmaceutical interventions (NPIs) might be the first-line of response against influenza infection [5]. Hence, NPIs are particularly critical for influenza control and prevention.

Influenza virus is transmitted rapidly in situations where many people gather in enclosed spaces and in close proximity such as subways, trains, airplanes, and ships [6]. Accordingly, school is a location where students gather together and influenza virus could transmit easily. Students, especially younger children, play a critical role in the transmission of acute respiratory infections within a community [7]. When influenza outbreaks occur, several NPIs can be implemented, including school closure (school is closed, and both students and staff stay at home), class dismal (lessons are suspended but school remains open with staff), class-suspension (lessons are suspended if members of the class have been found to have influenza symptoms, and students in this class have to go back to home and isolate), and symptom-based isolation (students who have influenza symptoms are quarantined at home) [8]. However, these noted NPIs lack sufficient effectiveness evidence and it is unknown whether these measures could be effective at controlling influenza outbreaks in school settings.

Therefore, our study directly addresses this gap by using a mathematical model to test the effectiveness of several NPIs, focusing on **class-suspension** and **symptom-based isolation**. Class suspension involves closing specific classes rather than the entire school. This means that if students in a class develop flu-related symptoms, lessons for that particular class will be suspended. Class-suspension interventions create a period of additional physical isolation between the students, and students typically learn at home when suspended from school. Symptom-based isolation means quarantine at onset andthe duration of the influenza symptoms. According to the ***guidelines for outbreak management of influenza-like cases (2018 version)***, enacted by China CDC (Centers for Disease Control and Prevention), class-suspension and isolation time is 7 days after the onset of fever symptoms or 48h after the disappearance of flu symptoms [9]. 

This study included two regions in southwest China as study places, including Zigong City in Sichuan Province and the Nanan district in the Chongqing municipality. The selected two areas have similar latitudes and longitudes (Zigong: 28°55’*N* − 29°38′N, 104°02′E − 105°16′E; Nan’an: 29°27′*N* − 29°37′N, 106°03′E − 106°47′E), both of which are located in a subtropical monsoon climate. When influenza broke out, **Zigong implemented the class-suspension policy**,** while Nanan engaged in symptom-based isolation.** Owing to the same latitude and similar climate in the two regions [10, 11], we purposively selected these two regions as study places to compare the effectiveness of two NPIs (class-suspension and symptom-based isolation) on influenza spread among children in primary school or kindergarten.

## Methods

### Data sources and participants

All influenza cases were under the China Communicable Disease Surveillance System (CCDSS). Participants included children in kindergartens and primary schools who were met the inclusion and exclusion criterion were purposively sampled.

#### Inclusion criterion

This study only included nursing school and primary school students, and these schools in Zigong City and Nanan District only have kindergarten students and primary students, respectively; diagnosed with influenza by level II and above hospitals; and whose infection information was reported to Communicable Disease Surveillance Network System of CDC.

#### Exclusion criterion

Students infected with COVID-19 or another upper respiratory infection; students or their parents unwilling to participate in this study.

### Definition of influenza-like illness (ILI)

Fever (body temperature ≥ 38℃), accompanied by either cough or sore throat. The onset of fever should occur within the current acute fever episode, and the determination of body temperature includes both the patient’s self-measured temperature and the temperature measured by medical institutions [9].

### ILI and influenza cases surveillance

Moving average method was used for influenza surveillance. To be specified: we predicted the current epidemic level of influenza using the average incidence in the past 5 years. We consider influenza activity when the actual prevalence in the current year is 25% higher than the predictable level. For the influenza epidemic, case surveillance was conducted in the community. Local hospitals are required to report case information (symptom onset and diagnosis time) through CCDSS within 24h after they identify influenza case. However, symptom-based surveillance was used in schools, we also monitor influenza through data on sick leave.

### Class suspension in different regions

The criterion for class suspension is different from Zigong and Nanan. Zigong requires suspension for a class for 4 days if any of the following conditions are met: (1) Five or more new influenza-like cases are reported in a class in one day; (2) The class has 30% or more influenza-like cases; (3) Two or more laboratory-confirmed influenza hospitalizations or deaths occur within a week. In contrast, Nanan only requires a 4-day suspension for confirmed influenza cases, and students with influenza-like symptoms must return to school only after symptoms have disappeared for 48 h.

When there is a rapid increase in influenza-like cases in primary and nursing schools in both regions, relevant prevention and control measures will be implemented if these cases are assessed as an outbreak. In Zigong, if a single school or kindergarten reports more than 30 influenza-like cases, or if there are more than 5 hospitalizations due to influenza-like illnesses within 1 week, the NPIs would be introduced [9]. In Nanan, if primary or kindergarten students have influenza-like symptoms or are diagnosed with influenza, NPIs will be introduced, regardless of the season or time.

### Epidemic dynamics model under different intervention measures

#### Class-suspension

**Fig. 1:**
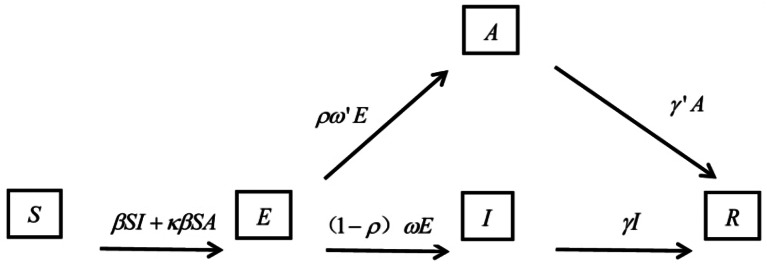
SEIAR model for class suspend measure

The SEIAR (susceptible-exposed-infectious/asymptomatic-removed) model (Fig. 1) was used as the class-suspension measure where only school factors were be considered in the model. Once students in a class were identified with influenza-related symptoms, the lessons of whole class were suspended, so the route of infection was cut off, resulting in an infection coefficient β = 0. In this model, asymptomatic students still have transmissibility, and class-suspension students still had risk of infection after they come back to school.

#### Symptom-based isolation

**Fig. 2:**
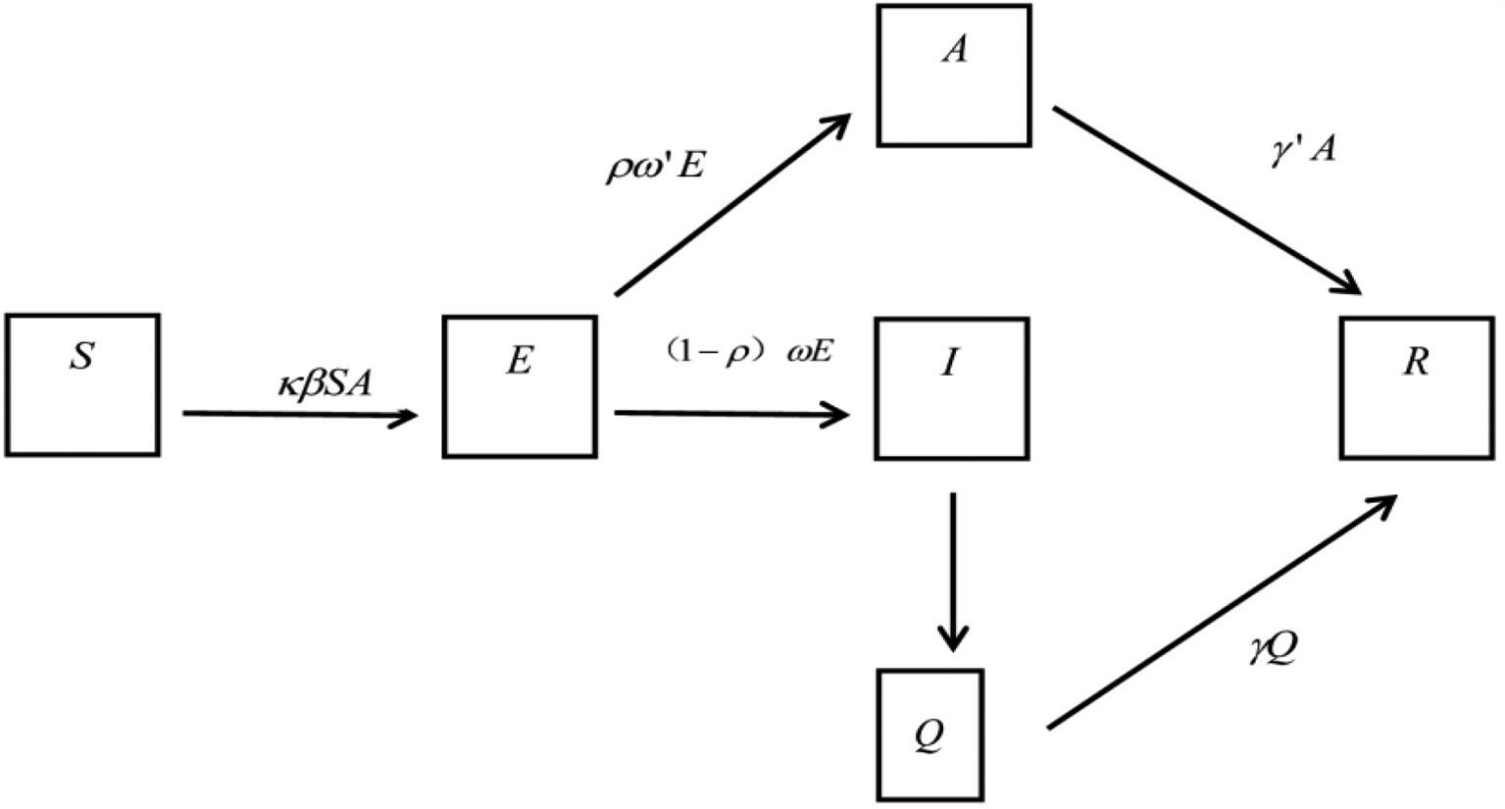
SEIAQR model for symptom-based measure

SEIAQR (susceptible-exposed-infectious/asymptomatic-quarantined-removed) model (Fig. 2) was used as the symptom-based isolation measure. Students who had influenza-related symptoms would be quarantined (Q), but the symptomatic students still had transmissibility.

#### Parameters of epidemic dynamics model

According to prior research [12–15], in above model, latent relative contagion coefficient k is 0.50, latency coefficient ω is 0.53, latent period coefficient is ω’ 0.83, latent infection rate p is 0.14, dominant coefficient of restitution γ is 0.21, latent coefficient of restitution γ’ is 0.24, infection coefficient β is calculated by epidemic data.

In addition, RT was used in this study to evaluate the real-time transmissibility. With an RT> 1, the epidemic of influenza will spread rapidly among people, and control measures should be strengthened and optimized. With an RT < 1, the epidemic of influenza is decreasing and the control measures are effective [16].

### Statistical analysis

WPS 2013 software was used to set up a database, R4.1.2 software was used to conduct statistical analysis and epidemic dynamics model building. EpiEstim package was used to fit RT, ggplot 2 package was used to draw plot. Normally distributed data were analyzed with analysis of t-test or variance; the Wilcoxon test was used for non-normally distributed data. All p values were based on two-sided statistical tests; statistical significance was defined as a *p* < 0.05.

## Results

### Baseline comparison

593 students in the Nanan District were included in this study and received symptom-based isolation. 1340 students in Zigong City were included in this study and received class-suspension. As Table 1 depicts, the vaccination rate of youth in Zigong City (21.27%) was statistically higher than the Nanan District (8.26%) *(p < 0.05)*. For parental educational level, there were more parents with higher levels of education (i.e., undergraduate, master’s degrees and above) in the Nanan District when compared with parents in Zigong City. This difference was statistically significant *(p = 0.025)*.

**Table 1:** The comparison of children’s baseline from Chongqing and Zigong

Variables		Nanan	Zigong	Total	χ^2^Z	*P*
Gender	Male	323 (54.47%)	673 (50.22%)	996	2.97	0.085
	Female	270 (45.53%)	667 (49.78%)	937		
School type	Kindergarten	69 (11.64%)	129 (9.63%)	198	1.8	0.179
	Primary school	524 (88.36%)	1211 (90.37%)	1735		
Vaccination(influenza vaccine tetravalent)	Inoculated	49 (8.26%)	285 (21.27%)	334	48.65	< 0.001
	Without inoculation	544 (91.74%)	1055 (78.73%)	1599		
Median time between symptoms onset and influenza diagnosis(day)		1.46 ± 1.66	1.50 ± 1.54	1933	-1.71	0.088
Parent’s educational level	Junior high school and below	76 (12.82%)	238 (17.76%)	314	9.33	0.025
	High school	147 (24.79%)	343 (25.6%)	490		
	Undergraduate	346 (58.35%)	719 (53.66%)	1065		
	Master and above	24 (4.05%)	40 (2.99%)	64		
Patient’s willingness of children’s vaccination	Accepted	429 (72.34%)	903 (67.39%)	1332	4.71	0.095
	No	27 (4.55%)	72 (5.37%)	99		
	Neutral	137 (23.1%)	365 (27.24%)	502		

### Incidence data comparison

**Fig. 3:**
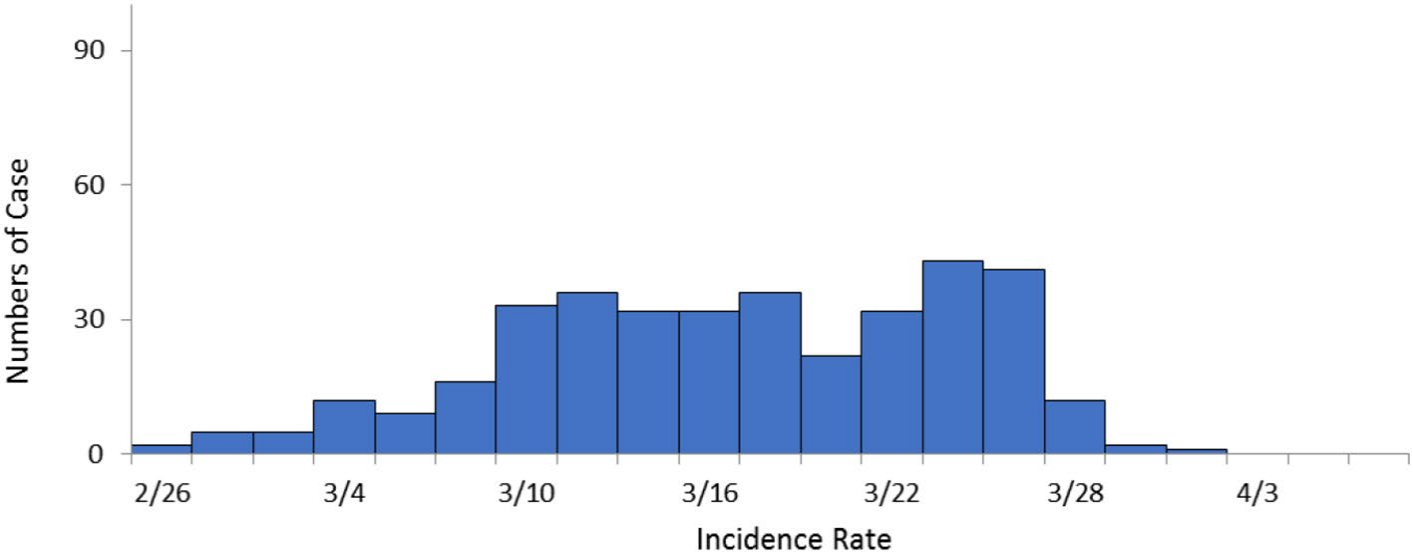
The incidence distribution of in Nanan

**Fig. 4:**
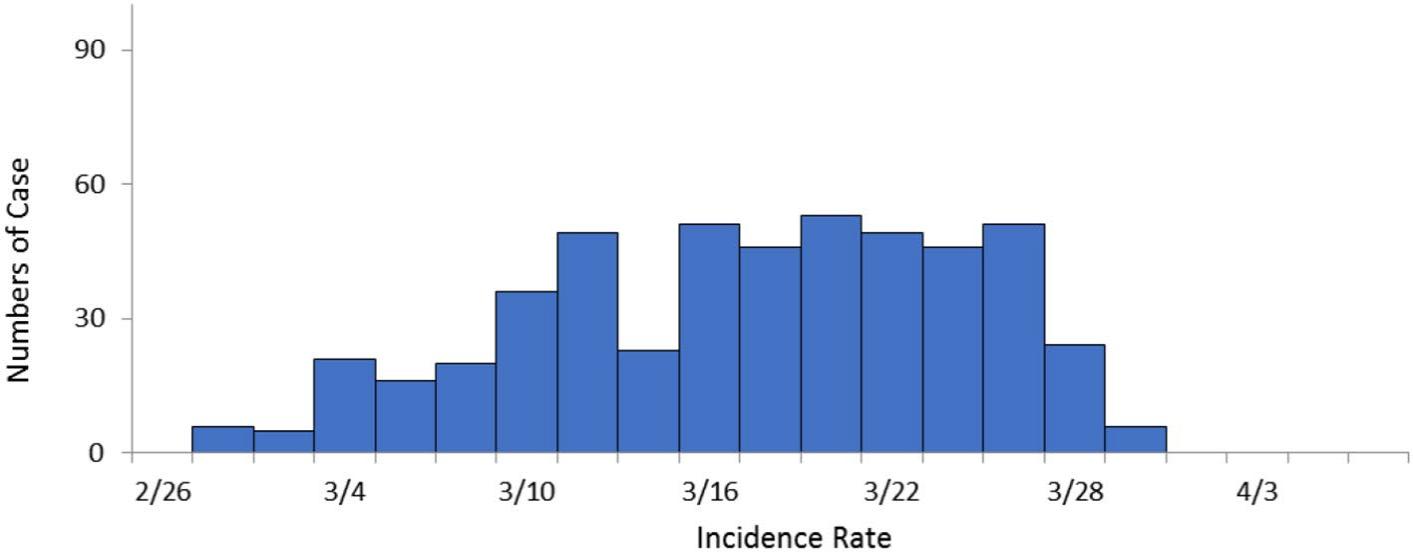
The incidence distribution of in Zigong

Results indicated that the outbreak time of influenza was from 26th, February to 31st, March in both Zigong City and the Nanan District. The incidence peak of the Nanan District was from 10th to 25th of March (Fig. 3). While the incidence peak of Zigong City was from 11th to 27th of March (Fig. 4).No statistical differences were observed for the epidemic trend between the Nanan District and Zigong City *(χ*^*2*^ *= 2.53*,* p = 0.112).*

### RT comparison

**Fig. 5:**
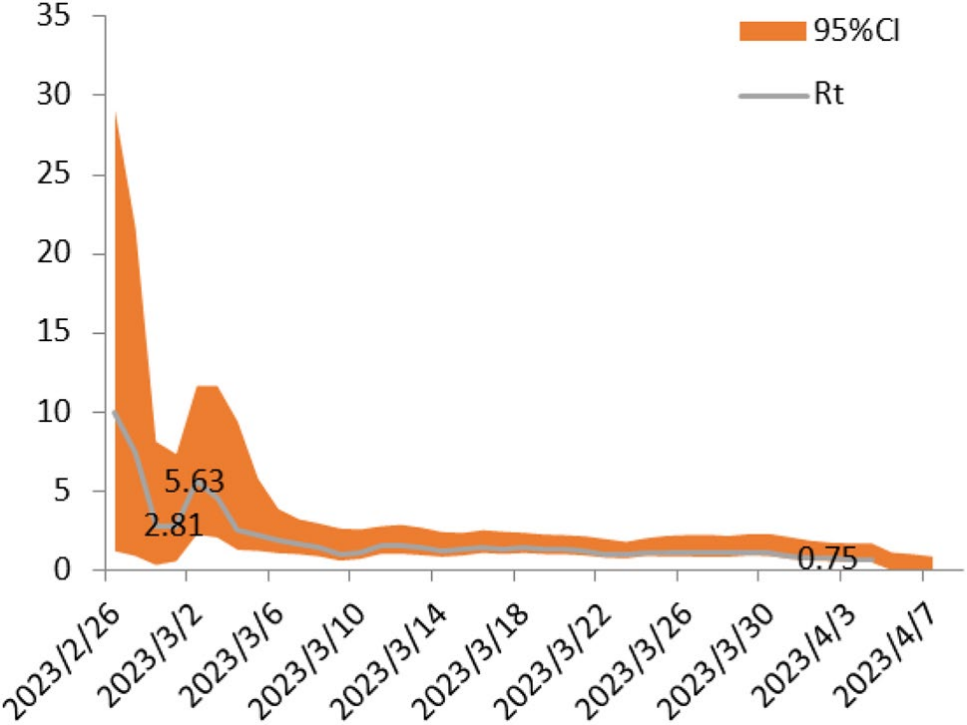
The variable of RT in Nanan

**Fig. 6:**
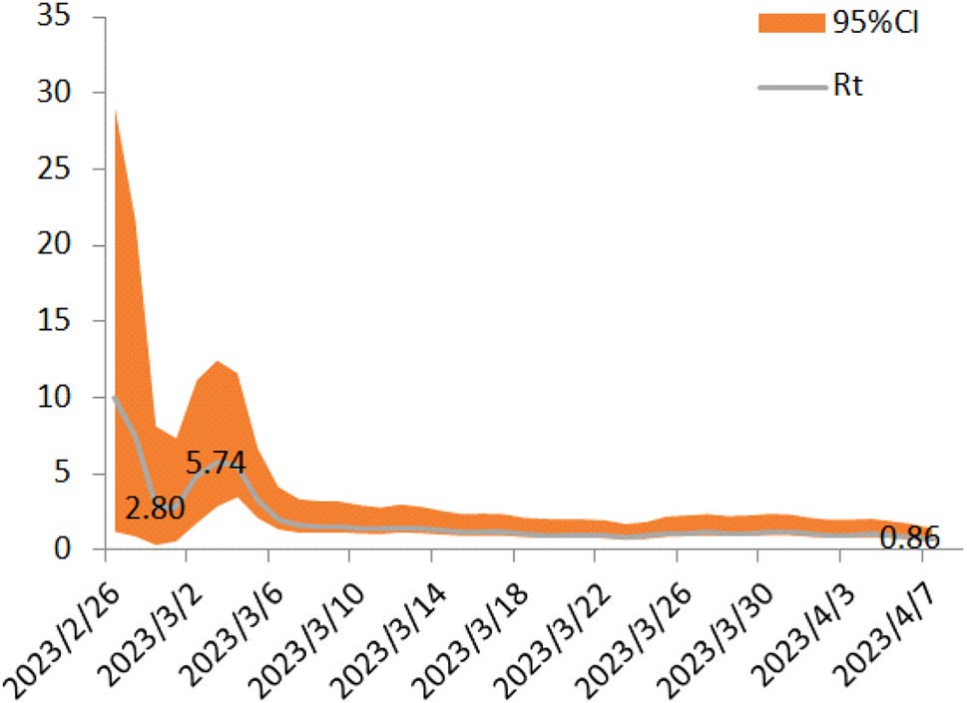
The variable of RT in Zigong

One primary school of similar enrollment in Zigong City and the Nanan District was purposively selected to compare the RT value. Differences were found in attack rate with Zigong City at 251/5060, which was similar to the Nanan District with 257/5960. Therefore, no significant differences were observed in attack rate *(P > 0.05)*. We exported the prevalence data of the two schools from 2/26/2023 to 4/7/2023 in Communicable Disease Surveillance Network System of CDC because of similar attack rates in both schools and similar timeline for the influenza outbreak duration. The change RT values in the Nanan District and Zigong City were depicted in Figs. 5 and 6, respectively. We compared the RT value and found no statistical significance in medium value of RT between the Nanan District (1.23) and Zigong City (1.16) (Table 2). Moreover, after quarantine and control measures were conducted, the RT value in the two regions was sharply decreased. Both the Nanan District and Zigong City decreased from 27th, February. The RT value dropped to < 1 in 24th, March and 2nd, April in Zigong City and the Nanan District, respectively.

**Table 2:** RT comparison between Nanan and Zigong

Variables	Nanan	Zigong	Z	*P*
RT value	1.23 ± 0.57	1.16 ± 0.59	-0.074	0.941

### ***The effects of NPIs on influenza incidence case***

The effects of NPIs on incidence cases of influenza were depicted in Table 3. The results indicate that the incidence cases after class-suspension was much higher than symptom-based isolation in both kindergarten and primary school, and the difference was statistically significant.

**Table 3:** The effects of NPIs on influenza incidence case (M (P_25_, P_75_)

NPIs	Kindergarten(*n* = 146)	Primary school(*n* = 68)	Total
Symptom-based isolation	4 (2,31)	30 (2,154)	7 (2,154)
Class-suspension for 7 days	10 (10,27)	92 (28,225)	61 (10,225)
Z	-1.97	-2.61	-9.38
P	0.049	0.009	< 0.001

### The effects of NPIs on attack rate

The effects of NPIs on attack rate were depicted in Table 4. The results revealed that the attack rate after class-suspension was much higher than symptom-based isolation in primary school, with a statistically significant difference. There was no statistically significant difference observed in kindergarten.

**Table 4:** The effects of NPIs on attack rate (Mean ± SD)

NPIs	Kindergarten(*n* = 146)	primary school(*n* = 68)	Total
Symptom-based isolation	2.19 ± 2.62	2.32 ± 2.33	2.25 ± 2.19
Class-suspension for 7 days	6.52 ± 1.25	4.63 ± 2.62	4.64 ± 2.43
Z	-1.67	-2.41	-0.54
P	0.095	0.016	0.586

## Discussion

Every year, influenza causes considerable disease burden for population health, especially for children, suggesting the importance of adopting effective measures to control the spread of influenza [17]. This study explored the effectiveness of two NPIs controlling influenza spread in primary school and kindergarten. According to the decrease of RT, we found that both symptom-based isolation and class-suspension are effective measures in the control of influenza spread, but which measure was more effective should be studied in future research. We further found the RT value below 1 was more quickly achieved in Zigong City (class-suspension) than the Nanan District (symptom-based isolation), while the overall attack rate in the two regions were similar. In all, the change of RT value of two NPIs were similar.

A meta-analysis conducted in 2014 indicated that school closure was a potential NPI during an influenza epidemic [18], but the costs of school closure are very expensive, and some parents are reluctant for class-suspension because they are worried that the learning for their children would be impacted. We found the symptom-based isolation and class-suspension were effective in controlling of influenza epidemic, and the RT value decreased fast after these measures were adopted. Moreover, the RT value in Zigong City declined more quickly than the Nanan District. On the one hand, it may be related to differences in vaccination rates, and on the other hand, it is related to class suspensions. Implementing measures to temporarily halt classes at the early stage of an influenza outbreak can effectively curb the rapid spread of the virus within school environments. This measure promptly isolates the sources of infection and potential infectors outside the school, allowing for rapid control of the flu outbreak in its early stages. After COVID-19, the incidence rates in the two regions had significantly increased compared to past five years, with widespread community transmission occurring (19–20). Under this influence, the “class suspension” measures in Zigong City during the subsequent handling of the pandemic resulted in an RT consistently around 1. During this period, the effectiveness of the “symptom-based isolation” measures in Nanan District was not much different; however, due to the implementation of “class suspension” in elementary schools, students often participated in recreational activities/tutoring outside. Because some parents of primary school children will send their children, who are on class-suspension, to after-school tutoring institutions to minimize disruptions in their learning. However, this practice might accelerate community transmission of influenza. In contrast, parents of kindergarten students pay more attention to their children’s health, as these children face less academic stress [21]. Therefore, we suggest that parents could reduce the frequency of sending children to after-tutoring institutions in influenza epidemic season and pay more attention to children’s health. In addition, we found that RT value stabilized in around 1 for a long time after the epidemic been controlled, which suggests that we should continue to adopt health surveillance to avoid repeated influenza epidemics. Also, health education is necessary to improve the parents’, students’ and teachers’ health literacy in influenza prevention and control.

### Limitations

Though effective measures were observed in our study, there were several limitations. First, we selected only two regions to compare the different NPIs in the control of influenza spread, and the sample size in Zigong City was two times larger than that in the Nanan District. This imbalance in sample size might contribute to sample bias. Second, in this study, we only focused on the RT value and the effectiveness of NPIs with prevalence, but we did not consider confounding factors such as the effects of influenza activity increased in the community. that might have influenced the effectiveness of NPIs. More factors that could have influenced the spread control should be investigated through further study.

## Conclusions

This study explored the effectiveness of two NPIs on control of influenza spread among children and it revealed that symptom-based isolation and class-suspension are effective measures. Symptom-based isolation was more effective for primary school. In addition, health education for influenza control and prevention for parents, teachers, and students is needed to improve health literacy and daily surveillance during the influenza season.

## Electronic supplementary material

Below is the link to the electronic supplementary material.

Supplementary Material 1

Supplementary Material 2

Supplementary Material 3

## Data Availability

The datasets generated and/or analyzed during the current study are available from the corresponding author on reasonable request.

## Abbreviations

ILI: Influenza-Like Illness
CCDSS: The China Communicable Disease Surveillance System
CDC: Centers for disease control and prevention
NPIs: Non-pharmaceutical interventions
RT: Effective reproduction number
SEIAR: Susceptible-exposed-infectious/asymptomatic-removed
SEIAQR: Susceptible-exposed-infectious/asymptomatic-quarantined-removed
