# Supplementary figures and images for: Non-pharmaceutical interventions (NPIs) to control influenza spread among children in primary school and kindergarten: class-suspension or symptom-based isolation?
Source: BMC Infect Dis. 2025 Mar 6;25:324. doi: 10.1186/s12879-025-10701-3 (PMC11887155; doi:10.1186/s12879-025-10701-3)

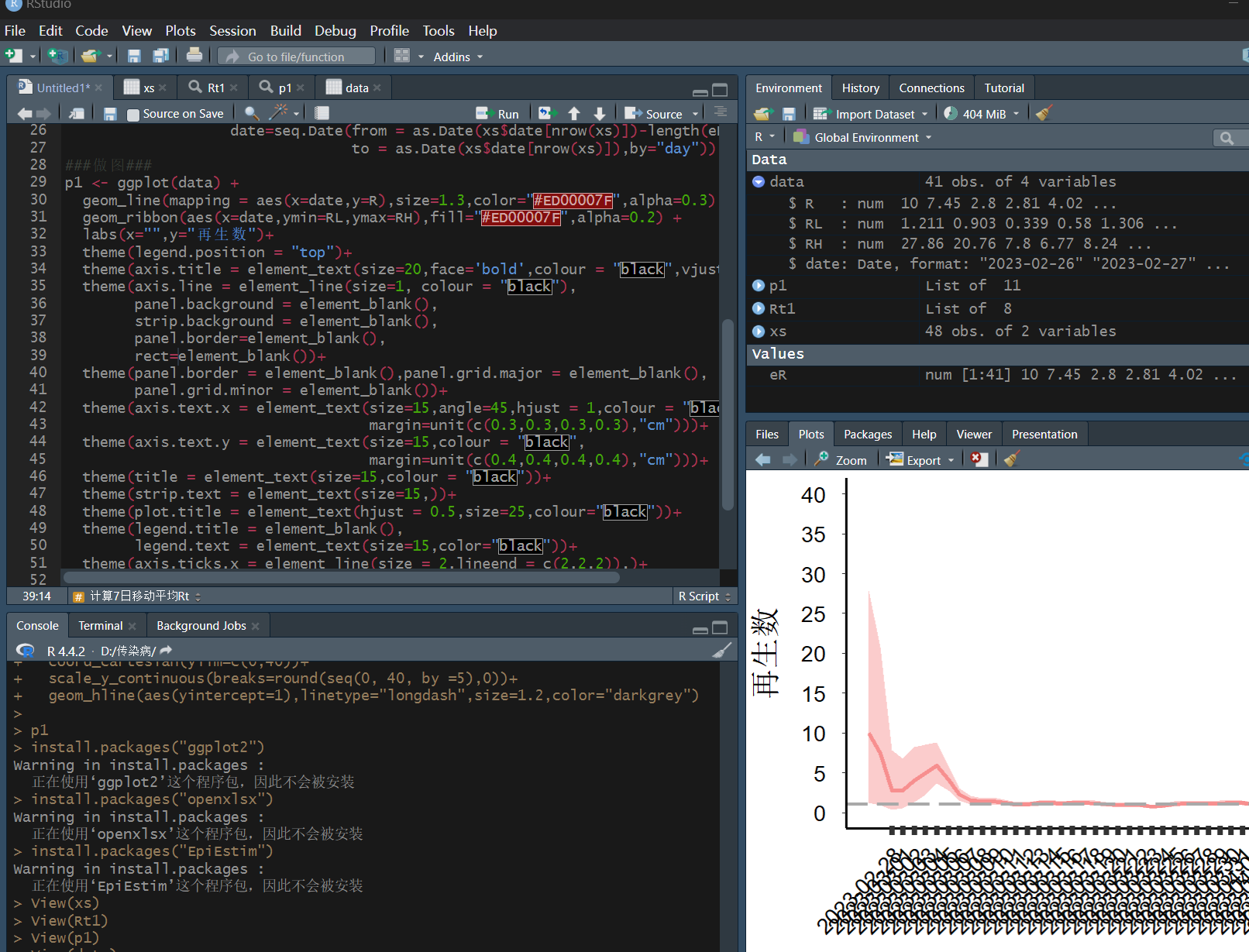

Supplement: Supplementary file 1 — Supplementary Material 1 [file 12879_2025_10701_MOESM1_ESM.png]
